# No Association between *HMOX1* and Risk of Colorectal Cancer and No Interaction with Diet and Lifestyle Factors in a Prospective Danish Case-Cohort Study

**DOI:** 10.3390/ijms16011375

**Published:** 2015-01-07

**Authors:** Vibeke Andersen, Tine Iskov Kopp, Anne Tjønneland, Ulla Vogel

**Affiliations:** 1 Organ Center, Hospital of Southern Jutland, 6200 Aabenraa, Denmark; 2 Institute of Regional Health Research, University of Southern Denmark, 5000 Odense, Denmark; 3 National Food Institute, 2860 Soborg, Denmark; E-Mail: tinis@food.dtu.dk; 4 Danish Cancer Society Research Center, 2100 Copenhagen, Denmark; E-Mail: annet@cancer.dk; 5 National Research Centre for the Working Environment, 2100 Copenhagen, Denmark; E-Mail: ubv@nrcwe.dk

**Keywords:** genetic epidemiology, colorectal cancer, heme iron, heme, meat intake, heme oxygenase, polymorphism, gene-environment interaction

## Abstract

Red meat is a risk factor for colorectal cancer (CRC). We wanted to evaluate whether a functional polymorphism in the *HMOX1* gene encoding heme oxygenase modifies risk of CRC or interacts with diet or lifestyle factors because this would identify heme or heme iron as a risk factor of CRC. The *HMOX1* A-413T (rs2071746) was assessed in relation to risk of colorectal cancer (CRC) and interactions with diet (red meat, fish, fiber, cereals, fruit and vegetables) and lifestyle (use of non-steroidal anti-inflammatory drug and smoking status) were assessed in a case-cohort study of 928 CRC cases and a comparison group of 1726 randomly selected participants from a prospective study of 57,053 persons. No association between *HMOX1* A-413T and CRC risk was found (TT *vs.* AA + TA; IRR = 1.15, 95% CI: 0.98–1.36, *p* = 0.10 for the adjusted estimate). No interactions were found between diet or lifestyle and *HMOX1* A-413T. *HMOX1* A-413T was not associated with CRC risk and no interactions with diet or lifestyle were identified in this large, prospective cohort with high meat intake. The results reproduced the previous findings from the same cohort and did not support a link between heme or heme iron and colorectal cancer. These results should be sought and replicated in other well-characterized cohorts with high meat intake.

## 1. Introduction

Heme oxygenase (HO-1, encoded by *HMOX1*) is the inducible, rate-limiting enzyme converting heme to iron, carbon monoxide (CO) and biliverdin which is subsequently converted into bilirubin. HO-1 activity reduces cellular oxidative stress by removing the pro-oxidant heme and at the same time produces pro-inflammatory iron. Thus, HO-1 activity may help discriminate the underlying mechanisms involved in meat-related colorectal cancer (CRC) carcinogenesis. The *HMOX1* A-413T and a microsatellite GT-dinucleotide repeat (GT)_n_ polymorphisms are in strong linkage and haplotype analyses indicated that the *HMOX1* A-413T genotype was likely to be the biologically functional polymorphism by modifying the promoter activity [1]. *HMOX1* polymorphisms have been found to be associated with various diseases and interactions with diet has been suggested [2]. We have previously evaluated the *HMOX1* A-413T polymorphism in a prospective Danish study of 383 cases of CRC and a sub-cohort of 763 participants [3]. We now extended this study in a larger cohort with power to assess gene-environment interaction. The aim of the present study was to evaluate whether the *HMOX1* polymorphism modifies risk of CRC or interacts with diet or lifestyle factors in a larger updated study from the same cohort because this would identify heme or heme iron as risk factors for CRC.

## 2. Results

Baseline characteristics of the study participants are shown in Table 1. Median intake of fiber was statistically significantly lower and median intake of alcohol was statistically higher among cases compared to members of the sub-cohort. Also, a significantly lower proportion of the cases were past or current users of hormone replacement therapy compared to the members of the subcohort. The genotype distribution of the polymorphisms in the comparison group did not deviate from Hardy-Weinberg equilibrium (results not shown). The variant allele frequency in the sub-cohort was 0.41.

**Table 1:** Baseline characteristics of the study participants by selected demographic and established colorectal cancer risk factors.

Variable	Cases		Subcohort		IRR ^a^ (95% CI)
	*n* (%)	Median (5%–95%)	*n* (%)	Median (55%–95%)	
Total	928 (100)	-	1726 (100)	-	-
Sex	-	-	-	-	-
Men	521 (56)	-	922 (53)	-	-
Women	407 (44)	-	804 (47)	-	-
Age at inclusion (years)	-	58 (51–64)	-	56 (50–64)	0.97 (0.95–0.99)
BMI (kg/m^2^)	-	26.3 (20.7–34.0)	-	25.6 (20.5–32.9)	1.03 (1.00–1.07) ^d^
Food intake (g/day)	-	-	-	-	-
Alcohol ^b^	-	15.0 (1.2–71.6)	-	14.0 (1.1–65.3)	1.04 (1.01–1.07) ^e^
Dietary fiber	-	19.9 (10.6–32.7)	-	20.6 (10.7–34.1)	0.88 (0.80–0.97) ^f^
Red and processed meat	-	113.0 (47.1–233.4)	-	109.2 (41.7–236.1)	1.02 (0.99–1.05) ^g^
Smoking status	-	-	-	-	-
Never	277 (30)	-	571 (33)	-	1.00 (ref.)
Past	284 (31)	-	508 (29)	-	1.03 (0.87–1.22)
Current	367 (39)	-	647 (37)	-	1.08 (0.92–1.27)
NSAID use ^c^	-	-		-	-
No	643 (69)	-	1185 (69)	-	1.00 (ref.)
Yes	285 (31)	-	541 (31)		1.00 (0.87–1.15)
HRT use among women	-	-	-	-	-
Never	250 (61)	-	425 (53)	-	1.00 (ref.)
Past	50 (13)	-	124 (15)	-	0.68 (0.50–0.93)
Current	107 (26)	-	255 (32)	-	0.76 (0.61–0.96)

Values are expressed as medians (5th and 95th percentiles) or as fractions (%); ^a^ Incidence rate ratios (IRRs) for CRC-mutually adjusted; ^b^ Among current drinkers; ^c^ NSAID use is defined as ≥2 pills per month during one year; ^d^ Risk estimate per 2 kg/m^2^ increment of BMI; ^e^ Risk estimate for the increment of 10 g alcohol per day; ^f^ Risk estimate for the increment of 10 g dietary fibers per day; ^g^ Risk estimate for the increment of 25 g red and processed meat per day; BMI, Body mass index; NSAID, Non-steroidal anti-inflammatory drugs; HRT, Hormone replacement therapy; IRR, Incidence rate ratios; 95% CI, 95% confidence interval.

As shown in Table 2 no association between *HMOX1* A-413T and CRC risk was found (TA *vs.* AA; Incidence rate ratios (IRR) = 1.00% and 95% Confidence Interval (95% CI): 0.86–1.15, *p* = 0.94, TT *vs*. AA; incidence rate ratio (IRR) = 1.15, 95% CI: 0.95–1.38, *p* = 0.15, and TT *vs*. AA + TA; IRR = 1.15, 95% CI: 0.98–1.36, *p* = 0.10, respectively, for the fully adjusted estimates (Table 2). A potential recessive effect was found and therefore AA and AT carriers were grouped *vs.* TT carriers to maximize the statistical power.

**Table 2:** Incidence rate ratios (IRR) for colorectal cancer in relation to *HMOX1* A-413T (rs2071746).

HMOX1	*n*_cases_ (%)	*n*_sub-cohort_ (%)	IRR ^a^ (95% CI)	IRR ^b^ (95% CI)	*p*-Value ^c^
AA	310 (33)	587 (34)	1.00 (ref.)	1.00 (ref.)	-
TA	446 (40)	864 (50)	1.01 (0.87–1.16)	1.00 (0.86–1.15)	0.94
TT	172 (19)	275 (16)	1.16 (0.97–1.40)	1.15 (0.95–1.38)	0.15
TA + TT *vs.* AA	618 (67)	1139 (66)	1.04 (0.91–1.20)	1.03 (0.90–1.18)	0.64
TT *vs.* AA + TA	172 (19)	275 (16)	1.16 (0.98–1.37)	1.15 (0.98–1.36)	0.10

^a^ Crude-adjusted for age and sex; ^b^ In addition, adjusted for smoking status, alcohol, HRT status (women only), BMI, use of NSAID, intake of red and processed meat, and dietary fiber; ^c^ *p*-value for the adjusted estimates. IRR, Incidence rate ratios.

No interactions were found between diet and *HMOX1* A-413T using the continuous scale (*p*-value for interaction (P_int_); meat = 0.97, fish = 0.18, cereal = 0.58, fiber = 0.69, fruit = 0.57, vegetables = 0.14) (Table 3). This analysis describes the increased risk pr25 gram meat per day (Table 3). Similarly, no interactions with diet were found using the tertile analysis (Table 4). No interactions were found between life style and *HMOX1* A-413T (use of non-steroid anti-inflammatory drug *p* = 0.11, smoking status *p* = 0.75) (TT *vs*. AA + TA; IRR = 1.15, 95% CI: 0.98–1.36, *p* = 0.10 for the adjusted estimate) (Table 5 and Table 6).

**Table 3:** Interaction between dietary factors and *HMOX1* A-413T (rs2071746) in relation to colorectal cancer risk.

HMOX1	IRR ^a^ (95% CI)	IRR ^b^ (95% CI)	*p*-Value ^c^	IRR ^a^ (95% CI)	IRR ^b^ (95% CI)	*p*-Value ^c^
	Red and processed meat			Fish		
AA + TA	1.02 (0.99–1.05)	1.02 (0.99–1.05)	0.97	0.96 (0.90–1.03)	0.97 (0.91–1.04)	0.18
TT	1.02 (0.96–1.09)	1.02 (0.96–1.09)		0.85 (0.72–1.00)	0.86 (0.73–1.02)	
	Dietary cereal			Dietary fiber		
AA + TA	0.95 (0.90–1.00)	1.01 (0.94–1.09)	0.58	0.87 (0.79–0.97)	0.89 (0.80–0.99)	0.69
TT	0.98 (0.89–1.09)	1.04 (0.93–1.17)		0.84 (0.68–1.04)	0.85 (0.69–1.05)	
	Fruit			Vegetables		
AA + TA	0.97 (0.95–1.00)	0.98 (0.96–1.01)	0.57	1.00 (0.96–1.04)	1.04 (0.99–1.08)	0.14
TT	0.96 (0.92–1.01)	0.97 (0.93–1.02)		0.93 (0.85–1.02)	0.97 (0.88–1.06)	

^a^ Analysis adjusted for smoking status, alcohol, HRT status (women only), BMI, use of NSAID, intake of red and processed meat, and dietary fiber; ^b^ *p*-value for interaction between polymorphisms and dietary factors for the adjusted estimates; ^c^ *p*-value for the adjusted estimates. Red and processed meat per 25 g/day, Fish per 25 g/day, Dietary cereal per 50 g/day, Dietary fiber per 10 g/day, Fruit per 50 g/day, Vegetables per 50 g/day. IRR, Incidence rate ratios.

**Table 4:** IRR for colorectal cancer for tertiles of intake of dietary factors for *HMOX1* A-413T (rs2071746).

HMOX1	1. Tertile		2. Tertile		3. Tertile		1. Tertile	2. Tertile	3. Tertile	*p*-Value ^b^
	*n*_c_	*n*_s_	*n*_c_	*n*_s_	*n*_c_	*n*_s_	IRR (95% CI) ^a^	IRR (95% CI) ^a^	IRR (95% CI) ^a^	
Red and processed meat										
AA + TA	251	548	257	462	248	441	1.00 (ref.)	1.25 (1.05–1.50)	1.28 (1.06–1.56)	0.33
TT	57	98	55	101	60	76	1.32 (0.98–1.78)	1.21 (0.92–1.61)	1.52 (1.13–2.04)	
Dietary cereal										
AA + TA	252	421	259	514	245	516	1.00 (ref.)	0.91 (0.76–1.10)	0.96 (0.76–1.21)	0.51
TT	61	97	52	91	59	87	1.07 (0.80–1.42)	0.99 (0.72-1.35)	1.27 (0.92–1.75)	
Fruit										
AA + TA	253	452	252	460	251	539	1.00 (ref.)	1.00 (0.83–1.20)	0.92 (0.74–1.13)	0.93
TT	60	90	59	89	53	96	1.20 (0.90–1.60)	1.13 (0.85–1.50)	1.02 (0.74–1.51)	
Fish										
AA + TA	257	528	255	417	244	506	1.00 (ref.)	1.12 (0.94–1.33)	0.93 (0.77-1.12)	0.57
TT	58	87	55	86	59	102	1.32 (0.98–1.78)	1.21 (0.91–1.61)	1.01 (0.76-1.34)	
Dietary fiber										
AA + TA	253	436	253	437	250	578	1.00 (ref.)	0.97 (0.82–1.16)	0.81 (0.68–0.97)	0.37
TT	60	98	59	75	53	102	1.05 (0.79–1.40)	1.33 (1.01–1.76)	0.89 (0.65–1.18)	
Vegetables										
AA + TA	249	439	252	501	255	511	1.00 (ref.)	1.06 (0.88–1.27)	1.12 (0.91–1.37)	0.28
TT	64	78	57	97	51	100	1.35 (1.02–1.80)	1.21 (0.90–1.62)	1.09 (0.79–1.49)	

^a^ Analysis adjusted for smoking status, alcohol, HRT status (women only), BMI, use of NSAID, intake of red and processed meat, and dietary fiber; ^b^ *p*-value for interaction between polymorphisms and dietary factors for the adjusted estimates; _c_ cases, _s_ sub-cohort. Tertiles of red and processed meat (<94.2266 g, 94.2266 g < and <139.266 g, >139.266 g), fish (<30.0189 g, 30.0189 g < and <48.0483 g, >48.0483 g), dietary fiber (<17.2846 g, 17.2846 g < and <22.4505 g, >22.4505 g), cereals (<152.202 g, 152.202 g < and <218.014 g, >218.014 g), fruit (<115.622 g, 115.622 g < and <224.174 g, >224.174 g), vegetables (<113.755 g, 113.755 g < and <197.250 g, >197.250 g). IRR, Incidence rate ratios.

**Table 5:** Interaction between NSAID use and *HMOX1* A-413T (rs2071746) in relation to colorectal cancer risk.

HMOX1	NSAID Use *n*_c_/ *n*_s_		NSAID Use IRR (95% CI)^a^		NSAID Use IRR (95% CI) ^b^		*p*-Value ^c^
	No	Yes	No	Yes	No	Yes	
AA + TA	539/1004	217/447	1.00 (ref.)	0.95 (0.81–1.11)	1.00 (ref.)	0.94 (0.80–1.10)	0.11
TT	104/181	68/94	1.05 (0.85–1.30)	1.32 (1.03–1.70)	1.04 (0.84–1.29)	1.30 (1.01–1.66)	

^a^ Crude-adjusted for age and sex; ^b^ In addition, adjusted for smoking status, alcohol, HRT status (women only), BMI, intake of red and processed meat, and dietary fiber; ^c^ *p*-value for interaction for the adjusted estimates; _c_ cases, _s_ sub-cohort. IRR, Incidence rate ratios.

**Table 6:** Interaction between smoking status and *HMOX1* A-413T (rs2071746) in relation to risk of colorectal cancer

HMOX1	Never	Past	Current	Never	Past	Current	Never	Past	Current	*p*-Value ^c^
	*n*_c_/ *n*_s_	*n*_c_/ *n*_s_	*n*_c_/ *n*_s_	IRR (95% CI) ^a^	IRR (95% CI) ^a^	IRR (95% CI) ^a^	IRR (95% CI) ^b^	IRR (95% CI) ^b^	IRR (95% CI) ^b^	
AA + TA	228/484	232/416	296/551	1.00 (ref.)	1.07 (0.89–1.28)	1.12 (0.94–1.33)	1.00 (ref.)	1.06 (0.88–1.27)	1.08 (0.90–1.28)	0.75
TT	49/87	52/92	71/96	1.24 (0.90–1.69)	1.11 (0.83–1.50)	1.34 (1.02–1.75)	1.20 (0.88–1.64)	1.10 (0.82–1.48)	1.30 (0.98–1.71)	

^a^ Crude-adjusted for age and sex; ^b^ In addition, adjusted for alcohol, HRT status (women only), BMI, use of NSAID, intake of red and processed meat, and dietary fiber; ^c^ *p*-value for interaction for adjusted risk estimates; _c_ cases, _s_ sub-cohort. IRR, Incidence rate ratios.

## 3. Discussion

In the present candidate gene study, we extended our previous study of *HMOX1* A-413T polymorphism in relation to diet, lifestyle and colorectal carcinogenesis in a study group of 383 CRC cases and 763 members of the comparison group to a larger cohort encompassing 928 number of cases and 1726 members of the comparison group and included more dietary factors. We found no association between *HMOX1* A-413T and CRC risk and no interactions between diet and lifestyle and *HMOX1* A-413T. Although no statistically significant interactions were found, we found that meat intake was associated with increased risk of CRC among carriers of both genotypes. Also, fiber intake was associated with lowered risk of CRC among carriers of both genotypes. We thus reproduced the previous finding that the studied *HMOX1* polymorphism was not associated with risk of CRC [3]. Our analyses suggest that genetically determined variation in heme oxygenase activity does not modify risk of CRC. Furthermore, we observed no interaction with meat intake. Thus, the results not support that intake of heme or heme iron from meat is linked to CRC. The investigated *HMOX1* A-413T polymorphism was selected based on the role of the gene product in heme metabolism and the functional effect of the polymorphism. Of the two strongly linked *HMOX1* promoter polymorphisms, the (GT)_n_ repeats and the A-413T, the A-413T genotype was most likely responsible for the effect on the promoter activity [1]. Accordingly, this polymorphism has been found to be associated with various diseases [1,4,5]. To the best of our knowledge, apart from our previous analysis, no other large studies of heme oxygenase in relation to CRC have been performed to date [3].

The study design has several strengths. The study design used in this study is well suited for analyzing interactions between genes and meat intake due to the collection of dietary and life style factors before diagnosis, the cases and controls being drawn from the same population, the large sample size and the relatively high meat intake [3,6,7,8,9,10,11] which may contribute to differential association pattern among populations with high and low meat intakes [12]. Study limitation includes limited power for detecting interactions. This study has more than 92% to detect a dominant effect of 1.4 had it been there [13]. The average meat intake among controls was 109 g/day in the present study group, which is associated with a 10% increased risk of CRC. The present study has approximately 80% chance of detecting a 3-fold difference in effect of meat intake with a primary effect of meat intake of 0.1 and an allele frequency of 0.41 [14]. Intake of meat may be associated with other CRC risk factors such as low fiber intake. However, we have adjusted for known risk factors in this cohort to minimize potential confounding.

## 4. Experimental Section

### 4.1. Studied Subjects

The Diet, Cancer and Health Study is an ongoing Danish cohort study designed to investigate the relation between diet, lifestyle and cancer risk [15]. The cohort consists of 57,053 persons, recruited between December 1993 and May 1997. All the subjects were born in Denmark, and the individuals were 50 to 64 years of age and had no previous cancers at study entry. Blood samples and questionnaire data on diet and lifestyle were collected at study entry.

### 4.2. Follow-Up and Endpoints

Follow-up was based on population-based cancer registries. Between 1994 and 31 December 2009, 970 CRC cases were diagnosed. A sub-cohort of 1897 persons was randomly selected within the cohort. Of these, 213 with missing genotype data were excluded. All information on genotypes and diet and lifestyle factors was available for 928 CRC cases and 1726 sub-cohort members.

### 4.3. Dietary and Lifestyle Questionnaire

Information on diet, lifestyle, weight, height, medical treatment, environmental exposures, and other socio-economic factors were collected at enrolment using questionnaires and interviews. In the food-frequency questionnaire, diet consumption was assessed in 12 categories of predefined responses, ranking from “never” to “eight times or more per day”. The daily intake was then calculated by using FoodCalc [15]. The study has been described in details elsewhere [3,6,7,8,9,10,11,15,16,17,18,19].

Smoking status was classified as never, past or current. Persons smoking at least 1 cigarette daily during the last year were classified as smokers.

The lifestyle questionnaire included information on the frequency regarding of use of “Aspirin”, “Paracetamol”, “Ibuprofen”, or “Other pain relievers”. Based on all records, we classified study subjects according to use of “any NSAID” (≥2 pills per month during one year) at baseline.

### 4.4. Genotyping

Buffy coat preparations were stored at minus 150 °C until use. DNA was extracted as described [20]. *HMOX1* A-413T (rs2071746) was genotyped by KBioscience (KBioscience, Hoddesdon, UK) by PCR-based KASP™ genotyping assay (Available online: http://www.lgcgenomics.com/). To confirm reproducibility, genotyping was repeated for 10% of the samples yielding 100% identity. Laboratory staff was blinded for the status of the samples.

### 4.5. Statistical Analysis

Deviation from Hardy-Weinberg equilibrium was assessed using a Chi square test.

Incidence rate ratios (IRR) and 95% Confidence Interval (95% CI) were calculated according to the principles for analysis of case-cohort studies using an un-weighted approach [21]. Age was used as the time scale in the Cox regression models. Tests and confidence intervals were based on Wald’s tests using the robust estimate of the variance-covariance matrix for the regression parameters in the Cox regression models [22] as previously described test [3,6,7,8,9,10,11,15,16,17,18,19].

All models were adjusted for baseline values of suspected risk factors for colorectal cancer such as body mass index (BMI) (kg/m^2^, continuous), NSAID (yes/no), use of hormone replacement therapy (HRT) (never/past/current, among women), smoking status (never/past/current), intake of dietary fiber (g/day, continuous), and red meat and processed meat (g/day, continuous). Cereals, fiber, fruit and vegetables were also entered linearly. All analyses were stratified by gender, so that the basic (underlying) hazards were gender specific. IRR was calculated separately for heterozygous and homozygous variant allele carriers. Since recessive effects were observed a recessive mode was used in the subsequent analyses.

We investigated possible interactions between the polymorphism and intake of meat, dietary fiber, cereals, fish, fruit, vegetables, smoking status and use of non-steroid anti-inflammatory drug (NSAID) using the likelihood ratio test [3,6,7,8,9,10,11]

In another set of interaction analyses between the polymorphism and the dietary intake subdivided in tertiles, dietary intake was entered as a categorical variable. Tertile cut-points were based on the empirical distribution among cases. The possible interactions were investigated using the likelihood ratio test.

The procedure PHREG in SAS (release 9.3; SAS Institute, Cary, NC, USA) was used for the statistical analyses. A *p* < 0.05 was considered to be significant.

The power to detect a dominant effect with an odds ratio of 1.5 and 1.4 was more than 98% and more than 92%, respectively [13].

The power to detect interaction was calculated. The average meat intake among controls was 109 g/day in the present study group, which is associated with a 10% increased risk of CRC. The power was 80% to detect a 3-fold difference in effect of meat intake with a primary effect of meat intake of 0.1 and an allele frequency of 0.41 [14].

### 4.6. Ethics Statement

All participants gave verbal and written informed consent. The Diet, Cancer and Health study was approved by the National Committee on Health Research Ethics (journal nr. (KF) 01-345/93) and the Danish Data Protection Agency.

## 5. Conclusions

The results reproduced our previous findings and do not support a link between heme or heme iron and colorectal cancer. The result should be evaluated in prospective cohorts with high meat intake.
